# Survival and death causes of patients with giant cell arteritis in Western Norway 1972–2012: a retrospective cohort study

**DOI:** 10.1186/s13075-019-1945-4

**Published:** 2019-06-25

**Authors:** L. K. Brekke, B.-T. S. Fevang, A. P. Diamantopoulos, J. Assmus, E. Esperø, C. G. Gjesdal

**Affiliations:** 1 Hospital for Rheumatic Diseases, Haugesund, Norway; 2 Department of Clinical Science, University of Bergen, Bergen, Norway; 3 Bergen Group of Epidemiology and Biomarkers in Rheumatic Disease (BEaBIRD), Department of Rheumatology, Haukeland University Hospital, Bergen, Norway; 4 Martina Hansens Hospital, Bærum, Norway; 5 Centre for Clinical Research, Haukeland University Hospital, Bergen, Norway

**Keywords:** Vasculitis, Giant cell arteritis, Temporal arteritis, Survival, Mortality, Causes of death, Epidemiology

## Abstract

**Background:**

Our objective was to determine the survival and causes of death in a large and well-characterized cohort of patients with giant cell arteritis (GCA).

**Methods:**

This is a hospital-based, retrospective, observational cohort study including patients diagnosed with GCA in Western Norway during 1972–2012. Patients were identified through computerized hospital records using the International Classification of Diseases (ICD)-coding system. Medical records were reviewed. Patients were randomly assigned population controls matched on age, sex, and geography from the Central Population Registry of Norway (CPRN). Date and cause of death were obtained from the Norwegian Cause of Death Registry (NCoDR). The survival was analyzed using Kaplan-Meier methods with the Gehan-Breslow test and the causes of death using cumulative incidence and Cox models for competing risks.

**Results:**

We identified 881 cases with a clinical diagnosis of GCA of which 792 fulfilled the American College of Rheumatology (ACR) 1990 classification criteria. Among those fulfilling the ACR criteria, 528 were also biopsy-verified. Cases were matched with 2577 population controls. A total of 490 (56%) GCA patients and 1517 (59%) controls died during the study period. We found no difference in the overall survival of GCA patients compared to controls, *p* = 0.413. The most frequent underlying causes of death in both groups were diseases of the circulatory system followed by cancer. GCA patients had increased risk of dying of circulatory disease (HR 1.31, 95% CI 1.13–1.51, *p* < 0.001) but lower risk of dying of cancer (HR 0.56, 95% CI 0.42–0.73, *p* < 0.001) compared to population controls.

**Conclusions:**

We found no difference in the overall survival of GCA patients compared to matched controls, but there were differences in the distribution of underlying death causes.

## Background

Giant cell arteritis (GCA) is the most common systemic vasculitis in adults and may present as a relapsing inflammatory disease of the elderly [1, 2]. Neither pathogenesis nor etiology of GCA is fully understood, although much has been learned in recent years [3, 4]. Patients with GCA risk a number of disease-related complications including blindness and aortic aneurysms, yet therapeutic options are limited [2]. The current cornerstone of GCA treatment, glucocorticoids, has serious adverse effects, and the newer treatments, such as interleukin (IL)-6 antagonism, have so far unclear long-term safety [5–8]. The potential for decreased survival of GCA patients has been recognized, but very few robust epidemiological studies have investigated this. A systematic review and meta-analysis published in 2017 reported no difference in the long-term mortality of GCA patients at a population level, but increased mortality in hospital-based cohorts, particularly in the years immediately after GCA diagnosis [9]. A subsequent meta-analysis reported no difference in all-cause mortality, but a significantly increased risk of death due to cardiovascular disease (CVD) [10]. However, there was substantial heterogeneity among underlying studies, and most individual studies were limited by small sample sizes, possible misclassification bias, lack of well-matched control cohorts, and/or short periods of follow-up [8, 11–27]. We report a 41-year follow-up study of 881 clinically diagnosed GCA patients whose disease characteristics have been thoroughly verified. Patient outcomes were compared to those of a large cohort of matched population controls. Separate results for the subset of patients fulfilling the American College of Rheumatology (ACR) 1990 criteria and the subset of biopsy-verified cases are presented to optimize comparison with earlier reports. Thus, this study aims to clarify the survivorship following GCA diagnosis as well as the cause-specific mortality in GCA patients.

## Materials and methods

This is a retrospective cohort study including patients diagnosed with GCA in Bergen Health Area during 1972–2012. Our material represents a predominantly Caucasian referral cohort recruited from the three somatic hospitals in Bergen health area: Haukeland University Hospital, Haraldsplass Deaconess Hospital, and Voss Hospital. These hospitals provide specialist healthcare services to approximately 440,000 inhabitants in Hordaland county, a mixed rural and urban area in Western Norway [28]. Patients were identified through computerized hospital records using the International Classification of Diseases (ICD)-coding system. We collected data by reviewing medical records of all patients registered with the diagnosis of GCA following an outpatient visit or admission to any ward in one of the study hospitals between 1 January 1972 and 31 December 2012 (41-year period). Further details about the patient inclusion process have been published previously [29]. Every Norwegian is given a unique 11-digit identification number at birth or time of immigration, and patients were matched for age (date of birth ± 1 month), sex, and county of residence to 3 control subjects randomly selected from the Central Population Registry of Norway (CPRN). The controls were required to be alive at the time of GCA diagnosis for their matched case, and this date was defined as the start of the observation period for the control. The observation period ended when the patient died or when the study ended (31 December 2012). We excluded duplicate control subjects and control subjects which were also among the cases. Extensive demographic and clinical data were collected for the cases, but for population controls, we had no available information on potential risk factors, comorbid conditions, or other clinical data. Data on the registered deaths were obtained from the Norwegian Cause of Death Registry (NCoDR) to which the death of every Norwegian is mandatorily reported. NCoDR also receives information on the date of death, but not always the specific cause of death, of Norwegians whom have emigrated. ICD-based NCoDR records are electronically available from 1951, using ICD-8 in 1969–1985, ICD-9 in 1986–1995, and ICD-10 from 1996 until today. Death causes in NCoDR were coded manually until 2005 when the Automated Classification of Medical Entities (ACME) system was introduced [30]. ACME is an automated coding system, which selects the underlying cause of death according to internationally adopted rules. The underlying cause of death (UCOD) refers to the disease or injury that initiated the train of morbid events leading directly to death. In contrast, a contributory cause of death (CCOD) is a significant condition that unfavorably influences the course of the morbid process and thus contributes to the fatal outcome, but does not directly cause death [31]. The registration of deaths in NCoDR was complete for the entire study period. Variables received from the NCoDR included the date of death and ICD codes of the underlying and contributory causes of death. Diagnoses were grouped according to the European Shortlist for Causes of Death 2012 version (COD-SL-2012), which categorizes death causes into disease groups and allows comparison of disease codes used in ICD versions 8 thru 10.

### Statistical analysis

Descriptive statistics were used to characterize the sample. The *t* test was used for comparing continuous variables and the chi-square or Fisher’s exact test for comparing categorical variables. The overall cumulative survival in cases and controls was estimated using Kaplan-Meier plots with registered death as the event (outcome). Cumulative survival was compared using the Gehan-Breslow test. Follow-up time was estimated using the reverse Kaplan-Meier method. The risks of death due to specific causes (circulatory disease, cancer, infection, or “other”) were analyzed using Cox proportional hazard (PH) models based on cumulative incidence for competing risks. The significance level was set to 0.05. The computing was done using the Statistical Package for the Social Sciences (SPSS) software version 24 (IBM Corp, Armonk) and R software version 3.5 [32]. The graphics were created using Matlab 9.0 (Mathworks Inc., Natick).

## Results

### Case identification

We identified 881 patients (71% female, mean age 73.0 (SD 8.6) years) with a clinical diagnosis of GCA, of which 792 fulfilled the ACR 1990 classification criteria for GCA. Among those fulfilling the ACR criteria, 528 were also biopsy-verified based on positive temporal artery biopsy (TAB). Among the 89 patients with a clinical GCA diagnosis not fulfilling the ACR 1990 criteria, 53 (60%) could be classified as having GCA according to the expansion of the 1990 ACR criteria for GCA proposed by Dejaco et al., though these criteria have not yet been validated [33]. For the remaining 25 patients, the clinical GCA diagnosis was in agreement with the opinion of the study rheumatologist following a thorough chart review, and 11 of these were also biopsy-verified. Further details about the patient selection process have been published previously [29]. The CPRN performed the random selection of population controls matched to cases by age, sex, and county of residence. One patient lacked a Norwegian personal identification number and could not be allocated matched controls. The other 880 cases were each matched with 3 population controls. We excluded 26 randomly selected controls that were also among the cases and 37 individuals who were randomly selected as controls for more than one case. Thus, the final cohort of population controls consisted of 2577 individuals (of which 2314 were matched to the 792 patients fulfilling ACR 1990 criteria and 1584 were matched to biopsy-verified GCA patients). Two individuals (both cases) had emigrated from Norway prior to death. NCoDR had information on the date of death but not the cause of death for these individuals. They are included in the survival analysis but excluded from the cause-specific analyses. Core characteristics of the included cases and controls are presented in Table 1.

**Table 1:** Core characteristics of cases and controls

	Clinical diagnosis		ACR 1990 criteria fulfilled		Biopsy-positive	
	Cases	Controls	Cases	Controls	Cases	Controls
	*N* = 881	*N* = 2577	*N* = 792	*N* = 2314	*N* = 528	*N* = 1584
Age at onset^1^	73 (8.6)	73 (8.6)	73.1 (8.5)	73.1 (8.5)	73.5 (7.9)	73.5 (7.9)
Female^2^	626 (71.1)	1823 (70.7)	566 (71.5)	1647 (71.2)	378 (71.6)	1134 (71.6)
Urban^2^	538 (61.1)	1360 (52.8)	484 (61.1)	1216 (52.5)	329 (62.3)	839 (53.0)
Biopsy-positive^2^	537 (61.0)	–	528 (66.7)	–	528 (100)	–
Median observation time^3^	8 [3, 14]	7 [3, 12]	8 [3, 14]	7 [3, 11]	6 [3, 13]	9 [6, 13]
Number of deaths during observation^2^	490 (55.6)	1517 (58.9)	432 (54.5)	1335 (57.7)	292 (55.3)	952 (60.1)
Age at death^1^	83.6 (7.5)	84.7 (7.5)	83.8 (7.4)	84.6 (7.6)	83.8 (6.9)	84.7 (7.4)
Time to death^4^	12 (11, 13)	12 (11, 12)	12 (11, 13)	12 (11, 12)	12 (11, 13)	12 (11, 12)

*ACR* American College of Rheumatology, *CI* confidence interval, *IQR* interquartile range, *SD* standard deviation

^1^Mean (SD)

^2^*N* (%)

^3^Median [IQR]

^4^Median (95% CI)

### Overall survival

**Fig. 1:**
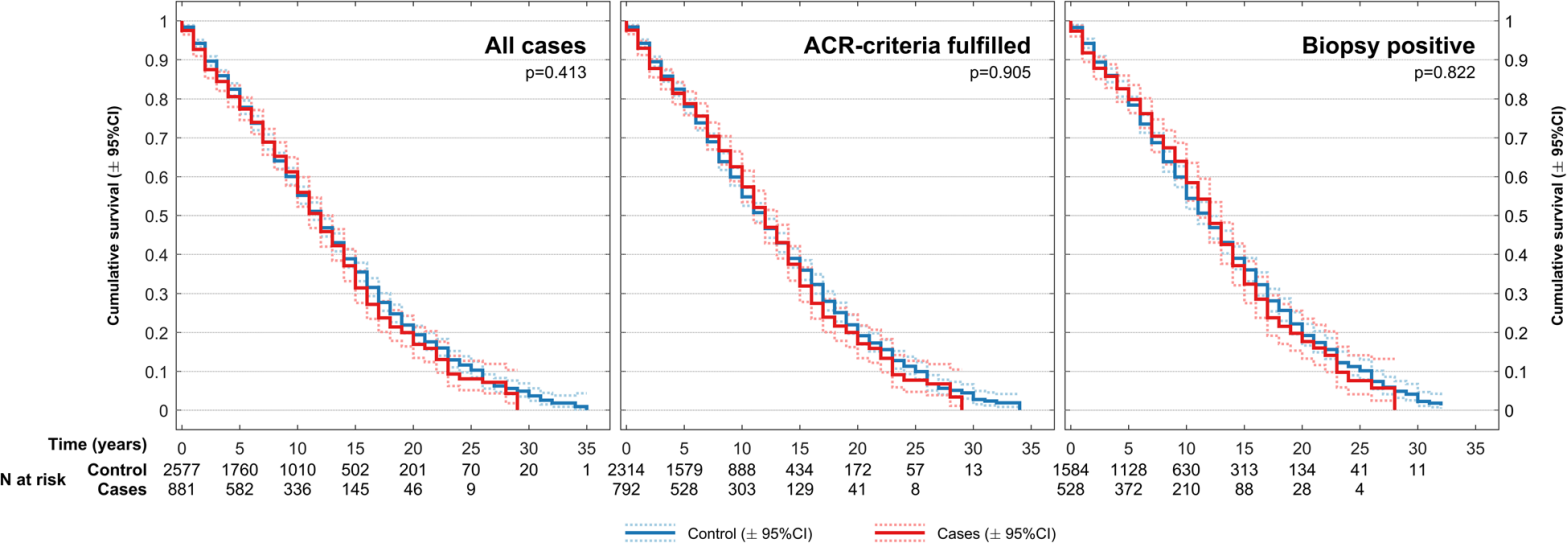
Kaplan-Meier survival plots for patients with GCA compared to matched controls

At the end of the study (31 December 2012), a total of 490 (69.6% female) GCA patients and 1517 (67.8% female) population controls were registered dead in NCoDR (Table 1). Mean age at death was 83.6 (SD 7.5) years for cases and 84.7 (SD 7.5) years for controls. Five years after disease onset, more than 80% of GCA patients were still alive, and at 10 years, approximately 50% were alive (Fig. 1). We found no significant difference in the overall cumulative survival or survival at any specific time point after diagnosis, for any subgroup of GCA patients compared to population controls (Fig. 1). Follow-up times ranged from 0 to 35 years with a median follow-up time of 8 years for cases and 7 years for controls (Table 1). Key features of our study compared to previous reports evaluating the survival of GCA cohorts are presented in Table 2.

**Table 2:** Key features of previous studies on survivorship in patients with giant cell arteritis (GCA)

Author and year of publication (Reference)	Time period and country	Study design	Inclusion criteria	Number of subjects (*N*)		Main conclusions—survival/mortality	
				Cases	Controls	Overall	Cause-specific
Lee 2018 [10]	1950–2010 Multi-national	Meta-analysis (8 studies included)	ACR 1990 or biopsy-proven	Pooled 1972 (877 deaths)	Expected rates	No difference	Increased CVD mortality
Hill 2017 [9]	1979–2015^a^ Multi-national	Meta-analysis (17 studies included)	ACR 1990 or biopsy-proven	Pooled 4733 (1853 deaths)	Expected rates	Excess mortality in hospital-based^b^	NR
Brekke (PS)	1972–2012 Norway	Retrospective, hospital-based	Clinical diagnosis	881 (490 deaths)	2577 matched	No difference	Increased CVD mortality, reduced cancer mortality
			ACR 1990	792 (432 deaths)	2314 matched		
			Biopsy-proven	528 (292 deaths)	1584 matched		
Aouba 2018 [13]	1980–2011 France	Retrospective, population-based	Death certificate listing GCA as a death cause ^c^	14,996 deaths	Expected rates	No difference	Increased CVD mortality, reduced cancer mortality
Li 2018 [14]	1990–2014 UK	Retrospective, population-based	Diagnosis of GCA in primary care database	9778 (3453 deaths)	92,268 matched	Excess mortality first 5 years, no difference long-term	NR
Catanoso 2017 [34]	1986–2013^d^ Italy	Retrospective, population-based	Biopsy-proven	285 (120 deaths)	285 matched	No difference	No difference
Baslund 2015 [35]	1993–2011 Denmark	Retrospective, population-based	Biopsy-proven	1787 (846 deaths)	33,953 matched	Excess mortality early and late after diagnosis	Increased CVD mortality, reduced cancer mortality
Mohammad 2015 [36]	1997–2010 Sweden	Retrospective, population-based	Biopsy-proven	840 (279 deaths)	Expected rates	Excess mortality first 2 years, no difference long term	NR
Kermani 2013 [37]	1950–2009^e^ USA	Retrospective, population-based	ACR 1990	204 (154 deaths)	Expected rates	No difference (except if LV manifestations)	Increased GIT mortality
Ninan 2011 [27]	1992–2006 Australia	Retrospective, population-based	Biopsy-proven	225 (71 deaths)	Expected rates	No difference	NR
Crow 2009 [15]	1991–2005 USA	Retrospective, hospital-based	Biopsy-proven	44 (21 deaths)	4400 matched	Excess mortality 5 years after GCA diagnosis	NR
Salvarani 2004 [12]	1950–1999 USA	Retrospective, population-based	ACR 1990	173 (NR)	Expected rates	No difference	NR
Uddhammar 2002 [16]	1973–1995 Sweden	Retrospective, hospital-based	Biopsy-proven (all fulfilled ACR1990)	136 (114 deaths)	Expected rates	Excess mortality (women only)	Increased CVD mortality
Gran 2001 [17]	1987–1997 Norway	Prospective, population-based	Biopsy-proven	64 (13 deaths)	256 matched	No difference	NR
Gonzalez-Gay 1997 [18]	1982–1996 Spain	Retrospective, population-based	Biopsy-proven	109 (22 deaths)	Expected rates	No difference	No difference
Matteson 1996 [19]	1981–1993 USA	Retrospective, hospital-based	ACR 1990	205 (49 deaths)	Expected rates	No difference	NR
Nesher 1994 [8]	1978–1992 Israel	Retrospective, hospital-based	Biopsy-proven or ACR 1990	43 (19 deaths)	Expected rates	Excess mortality (mainly first year)	NR
Rajala 1993 [20]	1969–1991 Finland	Retrospective, hospital-based	Biopsy-proven	66 (NR)	Expected rates	No difference (unless pre-existing CVD)	NR
Bisgård 1991 [11]	1973–1987 Denmark	Retrospective, hospital-based	Biopsy-proven	34 (18 deaths)	Expected rates	Excess mortality	NR
			Clinical diagnosis (probable)	146 (57 deaths)			
			Clinical diagnosis (possible)	85 (52 deaths)			
Nordborg 1989 [21]	1977–1987 Sweden	Retrospective, population-based	Biopsy-proven	284 (82 deaths)	Expected rates	No difference	Increased vascular mortality first year
Boesen 1987 [22]	1982–1985^f^	Prospective, population-based	Clinical diagnosis (including PMR)	46^g^ (5 deaths)	Expected rates	No difference	NR
Fjermestad 1983 [23]	1965–1980 Norway	Retrospective, hospital-based	Biopsy-proven	53 (14 deaths)	Expected rates	No difference	NR
Graham 1981 [24]	1968–1978 UK	Retrospective, hospital-based	Biopsy-proven	90 (32 deaths)	Expected rates	Excess mortality (women only)	NR
Jonasson 1979 [25]	1964–1977 Scotland	Retrospective, population-based	Biopsy-proven	124 (51 deaths)	Expected rates	No difference	No difference CVD mortality, NR other
Huston 1978 [26]	1950–1976^h^	Retrospective, population-based	Biopsy-proven or study-specific clinical criteria	42 (21 deaths)	Expected rates	No difference	No difference

*ACR* American College of Rheumatology, *CVD* cardiovascular disease, *GCA* giant cell arteritis, *GIT* gastrointestinal/digestive, *LV* large vessel, *NR* not reported, *PMR* polymyalgia rheumatica, *PS* present study, *UK* United Kingdom, *USA* United States of America

^a^Publication period

^b^Excess mortality in hospital-based cohorts only, not in population-based cohorts

^c^Underlying or contributing death cause

^d^Case inclusion thru 2012, registration of deaths extending thru 2013

^e^Case inclusion thru 2004, registration of deaths extending thru 2009

^f^Case inclusion thru 1982, registration of deaths extending thru 1985

^g^15 of 46 patients were biopsy-positive

^h^Case inclusion thru 1974, registration of deaths extending thru 1976

### Cause-specific risk of death

**Fig. 2:**
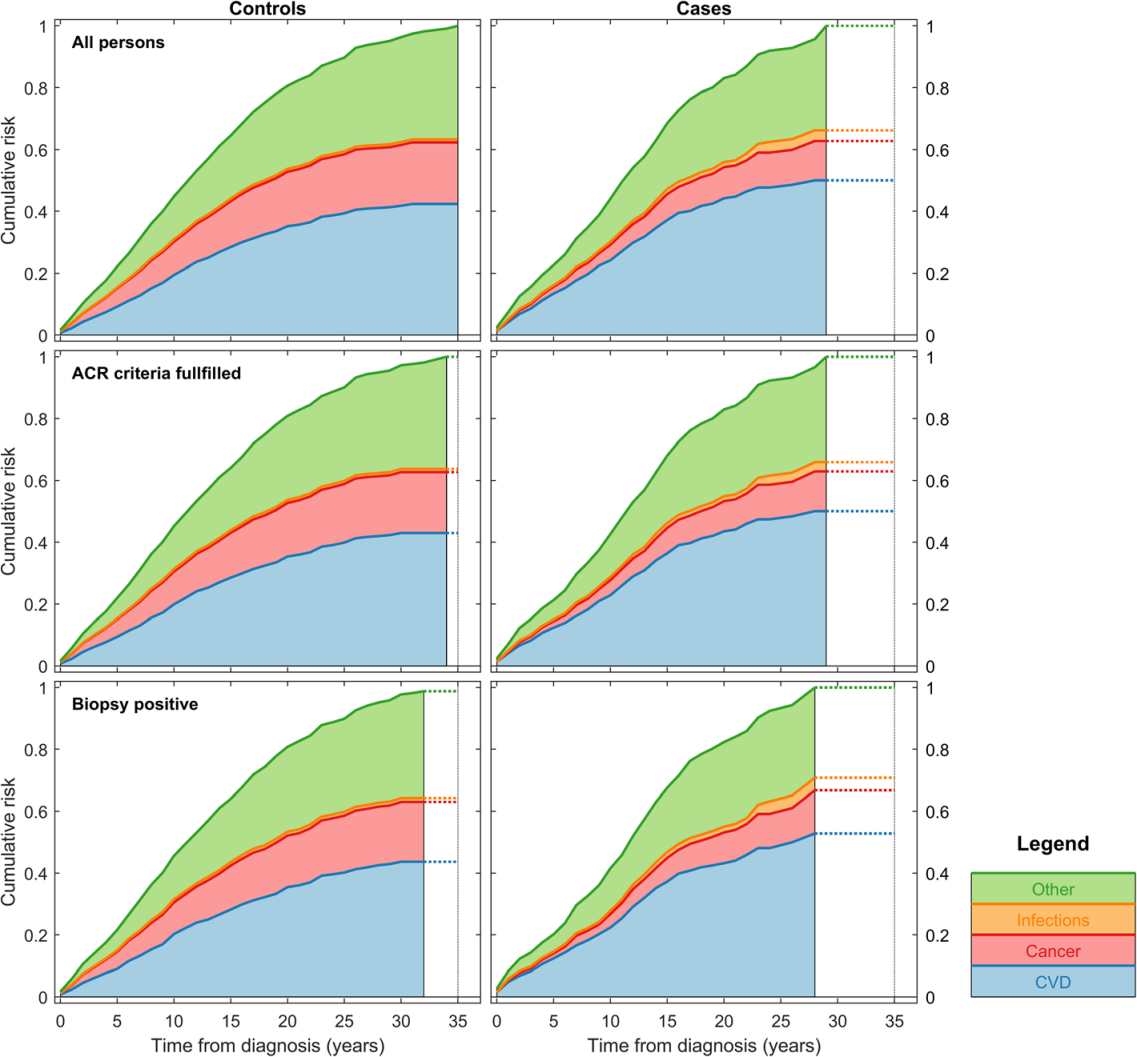
Kaplan-Meier plots for competing risks of death

**Fig. 3:**
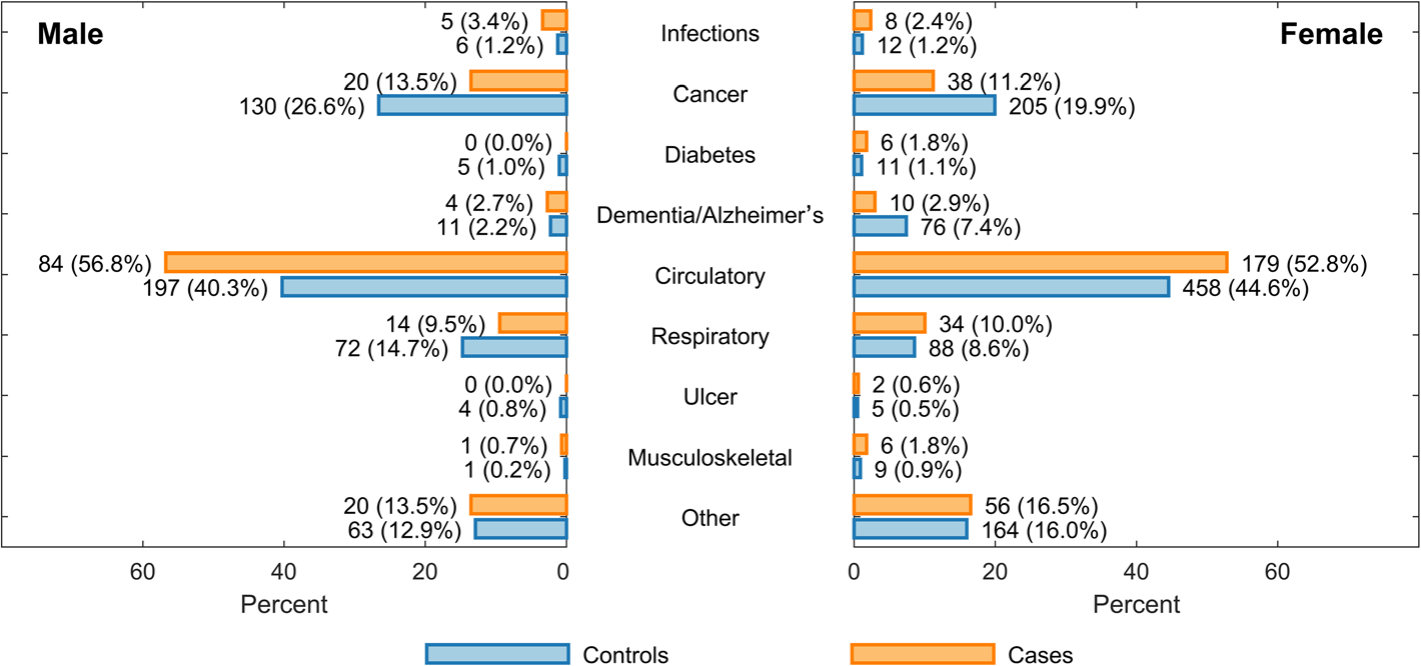
The distribution of underlying death causes in GCA patients and matched controls in Bergen Health Area 1972–2012 (all values represent the number (%) of persons with the registered death cause). Underlying death causes are grouped according to COD-SL-2012: infections—COD-SL-2012 codes 1.1–1.4; cancer—COD-SL-2012 codes 2.1.1–2.1.22; diabetes—COD-SL-2012 code 4.1; dementia/Alzheimer’s—COD-SL-2012 codes 5.1 and 6.2; circulatory disease—COD-SL-2012 codes 7.1–7.4; respiratory disease (including influenza and pneumonia)—COD-SL-2012 codes 8.1–8.4; ulcer—COD-SL-2012 code 9.1; musculoskeletal—COD-SL-2012 code 11; other—all other COD-SL-2012 codes. GCA, giant cell arteritis; COD-SL-2012, European Shortlist for Causes of Death (2012 version)

The most frequent UCODs in both GCA patients and matched controls were diseases of the circulatory system followed by cancer (Figs. 2 and 3). Combined, these diseases accounted for approximately two thirds of all deaths in both groups. The distribution differed between GCA patients and matched controls, with GCA patients having an increased risk of death due to circulatory disease (HR 1.31, 95% CI 1.13–1.51, *p* < 0.001) and infections (HR 2.34, 95% CI 1.15–4.80, *p* < 0.020) while having a lower risk of cancer deaths (HR 0.56, 95% CI 0.42–0.73, *p* < 0.001). As shown in Table 3, we observed the same for the ACR 1990 and biopsy-proven subgroups but with weaker, partially non-significant effects. We note that GCA itself was listed as UCOD or CCOD in very few patients despite a verified diagnosis of GCA (twice as UCOD and 10 times as CCOD, i.e., 0.4% and 2.0% of all registered deaths respectively). All underlying causes of death for cases and controls, grouped according to COD-SL-2012, are illustrated in Fig. 3.

**Table 3:** Cause-specific hazard ratios for the competing risks of death

	Events	HR	CI Lower	CI upper	*p* value
All cases, *N* = 3458					
CVD	918	1.31	1.13	1.51	0.0003
Cancer	393	0.56	0.42	0.73	0.0000
Infections	31	2.34	1.15	4.80	0.0197
Other	665	1.03	0.86	1.23	0.7770
ACR 1990 criteria fulfilled, *N* = 3106					
CVD	813	1.25	1.07	1.45	0.0047
Cancer	341	0.54	0.40	0.73	0.0001
Infections	28	2.03	0.95	4.34	0.0683
Other	585	1.04	0.86	1.26	0.6628
Biopsy-positive, *N* = 2112					
CVD	576	1.26	1.05	1.51	0.0140
Cancer	234	0.54	0.37	0.78	0.0010
Infections	24	1.94	0.85	4.44	0.1173
Other	410	0.98	0.78	1.24	0.8629

*CI* confidence interval, *CVD* cardiovascular disease, *HR* hazard ratio

### Circulatory diseases as the underlying cause of death

Among the 263 patients with circulatory disease as UCOD, 110 died of ischemic heart disease, 50 of other heart diseases, 58 of circulatory brain disease, and 45 of “other” circulatory diseases. Corresponding figures for controls were 655 deaths due to circulatory diseases, 308 deaths due to ischemic heart disease, 151 due to other heart diseases, 143 due to circulatory brain disease, and 53 due to other circulatory diseases. The COD-SL-2012 category “other circulatory disease” includes aneurysms and dissections, which are potential large vessel (LV) complications of GCA. However, the category “other circulatory disease” also includes embolism and thrombosis, rheumatic heart diseases, hypo- and hypertensive diseases, atherosclerosis, and pulmonary heart diseases, as well as other and unspecified disorders of the circulatory system. The numbers of registered deaths attributed to each of these diagnoses were small and our study lacked sufficient power to analyze the risk of these diagnoses separately.

## Discussion

In this study of 881 Norwegian GCA patients followed over a 41-year period, we found no difference in the overall survival of GCA patients compared to 2577 age-, sex-, and geographically matched controls. This is in agreement with several previous studies and supports the notion that a diagnosis of GCA does not negatively impact patients’ long-term survival [9, 10, 12, 13, 17–19, 21–23, 25–27, 34]. However, few previous reports have comprehensively described long-term competing risks of death in GCA patients. The results of our study indicate that GCA patients have an increased risk of death due to circulatory diseases and infections, but a decreased risk of death due to cancer over time. Below, we discuss factors that should further the understanding of the current evidence on survival and cause-specific mortality following GCA diagnosis.

Several factors may contribute to our finding of an equal long-term survival in GCA patients compared to that of population controls. Being monitored for a chronic disease such as GCA may represent a surveillance bias, in which concomitant diseases may be detected and treated earlier than they otherwise would have been. Also, it is possible that our included cases represent a subset of patients with more benign disease, in particular as large vessel (LV)-GCA may be underrepresented. Cases of LV-GCA may have been misdiagnosed or undiagnosed in the time period of our study, and their higher risk of mortality was therefore not captured by our study [38]. Specifically, there remains a gap in current knowledge concerning potential differences in the prognosis for cranial versus cranial plus LV manifestations. Despite increasing awareness of possible LV involvement, LV imaging is still often reserved for patients who present with large artery manifestations, rather than being used routinely in the evaluation of all patients diagnosed with GCA [2, 39]. Furthermore, the awareness of the true scope of LV manifestations is relatively recent. Therefore, published studies designed to analyze differences between LV and cranial subsets of GCA have had rather short periods of follow-up and thus decreased potential to detect differences in late-occurring outcomes such as death. Muratore et al. reported the hitherto largest study comparing patients with LV-GCA to those with cranial disease [40]. They included 120 patients with LV-GCA (defined by radiographic evidence of subclavian artery vasculitis) and 212 patients with cranial GCA (biopsy-positive) diagnosed between 1999 and 2008 and found that LV-GCA patients had higher relapse rate, greater corticosteroid requirements, and increased prevalence of aortic aneurysms. However, Muratore et al. did not compare differences in overall or cause-specific mortality. A recent publication by Macchioni et al. reported that large vessel involvement at diagnosis was associated with reduced survival (multivariate HR 5.14, 95% CI 1.34–19.74) in their retrospective Italian cohort, though acknowledging that inclusion of only TAB-positive patients in their study excluded patients with purely extracranial GCA from their analyses [41].

### Misclassification and selection bias

There is much heterogeneity among studies on GCA epidemiology, which calls for careful considerations when comparing results from different studies. Firstly, in the studies with a large sample size but unvalidated GCA diagnoses, there is a possibility of misclassification bias [13, 14]. In the inclusion process for our study, 35% of the initially selected patients coded as GCA were excluded to ensure a cohort of correctly diagnosed GCA [29]. Thus, studies of cohorts lacking validation of diagnosis may include a large number of misdiagnosed persons. Selection bias is another concern. We identified only two prospective survival studies and both concluded with no difference in overall mortality, but sample sizes were small, 64 and 46, and the inclusion criteria differed [17, 22]. In studies of later years, inclusion criteria have mainly been the fulfillment of ACR 1990 criteria for GCA or biopsy-proven cases only. Both of these approaches have limitations. Restriction to biopsy-proven cases has predominantly relied on TAB results and thus limited case selection to patients with cranial arteritis. Patients with LV involvement are less likely to have temporal artery abnormalities according to Muratore et al. who found that ACR classification criteria for GCA were satisfied in only 39% of LV-GCA patients compared to 95% of GCA patients with cranial arteritis (*p* < 0.001) [40]. The inclusion of only TAB-positive patients thus excludes a majority of LV-GCA patients. The ACR 1990 criteria are similarly flawed by the lack of incorporating modern imaging evidence of LV manifestations and by the narrow spectrum of clinical (mostly cranial) features included in the criteria [42, 43]. To minimize potential ascertainment bias in our study, we included patients given the diagnosis of GCA on clinical grounds. To allow for this, we thoroughly reviewed the medical records and we performed subgroup analyses restricted to ACR 1990 and biopsy-proven cases only to allow for comparison to other studies. Unfortunately, for the time period of our study, appropriate imaging tests for detection of LV-GCA would not have been performed in the majority of cases. A lack of complete capture of patients with LV-GCA is therefore a probable limitation of ours as well as most other hitherto published studies on GCA survival.

In the study by Aouba et al., only cases for which GCA was listed as an underlying or non-underlying cause of death in the death certificate were included [13]. We found that only 2.4% of those who died in our GCA cohort had GCA recorded as UCOD or CCOD on their death certificate. Thus, Aouba and colleagues have presumably not captured all GCA cases in their database, but the included cases may nevertheless be a representative sample. Their analysis of cause-specific death patterns yielded similar results to that of our study, finding an increased cardiovascular death risk but a decreased risk of death due to cancer.

### Timing of death after diagnosis

The first study to compare the survival of GCA patients to that of matched controls was published in 2009 [15]. The authors found an excess mortality in GCA patients at 5 years after GCA diagnosis, but also that the survival rates for cases and controls converged after approximately 11 years. Some other studies have reported similar findings [14, 36]. Baslund et al. analyzed death and causes of death in three time periods: 0–2, 2–10 and > 10 years after GCA diagnosis [35]. They argued that this subdivision reflects the clinical course of GCA, usually remitting within 6–24 months of disease onset but with a possibility of late involvement of the large vessels. Baslund and colleagues found an increased risk of death due to circulatory diseases during 0–2 years and > 10 years after the diagnosis of GCA. Increased vascular risk associated with GCA has also been reported by others and includes cardiovascular disease, thromboembolic disease, and LV complications [10, 13, 16, 21, 44, 45]. However, the underlying mechanisms of all the vascular risks are not entirely understood and may encompass both disease-related and treatment-related causes [46]. The possibility of incomplete capture of deaths due to late vascular complications is a limitation of every study with a short follow-up period. In contrast, the very long follow-up period in our study reduces the risk of missing late-occurring complications and diminishes the risk of an erroneous conclusion based on variations through time.

### Large vessel (LV) complications

LV complications were not a common cause of death in our GCA cohort. There were few registered deaths due to aortic aneurysm or dissection, and our study lacked sufficient power to analyze the risk of these diagnoses separately. Without autopsy data, there is a risk that deaths due to vascular complications might have been misclassified as caused by other (circulatory) disease. A large autopsy study by Östberg in 1971 found the prevalence of GCA to be higher than indicated by the clinical incidence and suggested that many cases remain clinically undiagnosed. Published reports indicate that GCA patients with LV manifestations have increased risk of death compared to GCA patients without LV manifestations and also compared to control individuals [37, 38]. Increasing use of modern imaging techniques allowing visualization of large vessels may improve the prognosis for these patients.

### Strengths and weaknesses

Our data are limited by the retrospective design and lack of data (for controls) on important risk factors such as smoking, use of medications, co-morbidities, and other potential confounders. We note that our cohort consists of cases with predominantly cranial GCA (> 60% with positive TAB). Thus, our results may not be representative for cases with purely extracranial GCA. A major strength is the well-defined cohort of GCA cases in our study resulting from a thorough review of clinical data, excluding misclassified cases, and including hospitalized patients as well as those only treated in outpatient clinics. The study also included a large cohort of population controls that were tightly matched with regard to the most significant of all risk factors for death—age. Access to national registries with mandatory reporting provided excellent completeness of data concerning dates and causes of deaths with virtually no loss to follow-up. The large sample size of both cases and controls rendered a well-powered analysis allowing us to detect relevant differences between the groups. Finally, the long inclusion period reduced the risk of evaluating random time variations, and the long follow-up period secured the inclusion of deaths due to late complications.

## Conclusions

Based on our findings, the long-term survival of GCA patients is comparable to that of population controls. The most frequent underlying causes of death in both GCA cases and controls were diseases of the circulatory system followed by cancer. However, GCA patients had increased risk of death due to circulatory diseases compared to controls. This should be emphasized in the management of patients with GCA, and contributing risk factors for circulatory death need to be further deciphered and appropriately targeted. We stress that our results may have limited transferability to patients with mainly extracranial disease. Improved understanding of the different subsets of GCA, specifically with or without LV involvement, and appropriate tailoring of treatment according to this, may alter the long-term outcomes for GCA also on a group level.

## Data Availability

Not applicable.

## Abbreviations

ACME: Automated Classification of Medical Entities
ACR: American College of Rheumatology
CCOD: Contributory cause of death
CI: Confidence interval
COD-SL-2012: European Shortlist for Causes of Death 2012 version
CPRN: Central Population Registry of Norway
CVD: Cardiovascular disease
GCA: Giant cell arteritis
GIT: Gastrointestinal
HR: Hazard ratio
ICD: International Classification of Diseases
IL: Interleukin
IQR: Interquartile range
LV: Large vessel
NCoDR: Norwegian Cause of Death Registry
NR: Not reported
PH: Proportional hazard
PMR: Polymyalgia rheumatica
PS: Present study
REC: Regional Ethics Committee
SD: Standard deviation
SPSS: Statistical Package for the Social Sciences
TAB: Temporal artery biopsy
UCOD: Underlying cause of death
UK: United Kingdom
USA: United States of America
